# Digitale Gesundheitsanwendungen in der Hals‑, Nasen- und Ohrenheilkunde

**DOI:** 10.1007/s00106-022-01271-w

**Published:** 2023-02-03

**Authors:** Benedikt Hofauer, Dominik Pförringer, Oliver Schöffski, Zhaojun Zhu, Christian Offergeld

**Affiliations:** 1 Klinik und Poliklinik für Hals‑, Nasen- und Ohrenheilkunde, Klinikum rechts der Isar, Technische Universität München, Ismaningerstr. 22, 81675 München, Deutschland; 2 Klinik und Poliklinik für Unfallchirurgie, Klinikum rechts der Isar, Technische Universität München, München, Deutschland; 3 Lehrstuhl für Gesundheitsmanagement, Universität Erlangen-Nürnberg, Nürnberg, Deutschland; 4 Klinik und Poliklinik für Hals‑, Nasen- und Ohrenheilkunde, Universitätsklinikum Freiburg, Freiburg im Breisgau, Deutschland

**Keywords:** Tinnitus, Schlafstörungen, Insomnie, Onkologie, Verhaltenstherapie, Tinnitus, Sleep disorders, Insomnia, Oncology, Cognitive behavioral therapy

## Abstract

**Hintergrund:**

Mit Verabschiedung des „Digitale-Versorgung-Gesetzes“ durch den Deutschen Bundestag Ende 2019 wurde unter anderem ermöglicht, dass digitale Gesundheitsanwendungen (DiGA) unter bestimmten Voraussetzungen von den gesetzlichen Krankenkassen erstattet werden können. Ziel dieser Arbeit ist die Identifikation von DiGA mit Bezug zur HNO-Heilkunde und die Beschreibung der zugrunde liegenden Evidenz.

**Material und Methoden:**

Es erfolgte eine Analyse des DiGA-Verzeichnisses nach DiGA, deren Indikationsbereich eine Erkrankung aus dem HNO-Bereich betrifft. Es wurden DiGA eingeschlossen, die entweder dauerhaft oder vorläufig aufgenommen wurden oder aktuell gestrichen sind, wenn hierzu weitere Informationen vorlagen. Es erfolgte eine Bewertung der zugrunde liegenden Evidenz nach den Empfehlungen des Oxford Centre for Evidence-Based Medicine für therapeutische Studien.

**Ergebnisse:**

Insgesamt wurden sechs DiGA mit direktem oder indirektem Bezug zur HNO-Heilkunde identifiziert, von denen drei dauerhaft und zwei vorläufig in das Verzeichnis aufgenommen wurden. Eine DiGA ist aktuell vom Hersteller zurückgezogen worden. Den dauerhaft aufgenommenen DiGA liegt eine Evidenz Grad 1b zugrunde.

**Schlussfolgerung:**

Die Einführung von DiGA wird teilweise auch kritisch diskutiert, dennoch stellt ihre Einführung einen innovativen Ansatz dar, und gerade für den HNO-Bereich sind bereits verschiedene DiGA mit hoher zugrunde liegender Evidenz verfügbar.

## Abstract (en)

**Background:**

With the passing of the “Digital Care Act” by the German Bundestag at the end of 2019, it was made possible, among other things, for digital health applications to be reimbursed by statutory health insurance companies under certain conditions. The aim of this work is to identify digital health applications related to ear, nose, and throat medicine and to describe the underlying evidence.

**Materials and methods:**

The digital health applications register was analyzed to identify digital health applications with an indication area relating to an otorhinolaryngologic disease. Digital health applications were included that were either permanently or provisionally included, or currently deleted if further information was available. The underlying evidence was assessed according to the recommendations of the Oxford Center for Evidence-Based Medicine for therapeutic studies.

**Results:**

A total of six digital health applications with a direct or indirect connection to otorhinolaryngology were identified, three of which were permanently and two provisionally included in the directory. One digital health application has currently been withdrawn by the manufacturer. The permanently recorded digital health applications are based on evidence level 1b.

**Conclusion:**

The introduction of digital health applications is sometimes also discussed critically, but they represent an innovative approach and various digital health applications with a high level of underlying evidence are already available, especially for the ear, nose, and throat area.

Schon vor Beginn der Corona-Pandemie sah sich die medizinische Versorgung in Deutschland mit unterschiedlichen Herausforderungen konfrontiert: In den nächsten Jahren werden viele ältere ÄrztInnen in den Ruhestand gehen, viele Praxen, gerade in ländlicheren Regionen, können bereits jetzt nicht mehr nachbesetzt werden. Gleichzeitig nimmt die Bereitschaft zu 60-Stunden-Wochen ab, und sogar die selbstständige Tätigkeit in einer eigenen Praxis ist heute weniger erstrebenswert – viele jüngere Kolleginnen und Kollegen entscheiden sich heutzutage für ein Angestelltenverhältnis mit einem geringeren Risiko, auch flexiblere Arbeitszeitmodelle und Teilzeitarbeit finden vermehrt Verbreitung. Der demografische Wandel wird zudem dazu führen, dass immer weniger ÄrztInnen für mehr ältere Menschen zuständig sein werden. Gleichzeitig werden jedoch mit den jüngeren ÄrztInnen immer mehr sog. Digital Natives berufstätig – also die Jahrgänge ab den 1980er-Jahren – für die viele der Herausforderungen des Gesundheitswesens durch eine stärkere Digitalisierung und Vernetzung gelöst werden können [8, 9].

Natürlich findet die Digitalisierung Einzug in das Gesundheitswesen, deutlich beschleunigt wurde dieser Prozess jedoch mit Beginn der Corona-Pandemie, beispielsweise hat in diesem Zusammenhang die Anzahl der durchgeführten Videosprechstunden von weniger als 3000 im Jahr 2019 auf 2,67 Mio. im Jahr 2020 zugenommen, und der Verabschiedung des „Digitale-Versorgung-Gesetzes“ durch den Deutschen Bundestag Ende 2019. Diese Initiative soll eine Grundlage für Innovationen auf unterschiedlichen Bereichen der digitalen Medizin schaffen – so wurden in diesem Zusammenhang auch digitale Gesundheitsanwendungen (DiGA) eingeführt, die unter bestimmten Voraussetzungen auch von den gesetzlichen Krankenkassen erstattet werden können. Bei DiGA handelt es sich um CE-gekennzeichnete Medizinprodukte, die folgende Eigenschaften erfüllen müssen [2, 14, 16]:

- Medizinprodukt der Risikoklasse I oder IIa
- Hauptfunktion beruht im Wesentlichen auf digitalen Technologien.
- Die digitale Hauptfunktion ist für die Erreichung des medizinischen Ziels ausschlaggebend.
- Ziel ist es, die Erkennung, Überwachung, Behandlung oder Linderung von Krankheiten oder die Erkennung, Behandlung, Linderung oder Kompensierung von Verletzungen oder Behinderungen zu unterstützen.
- Die DiGA wird entweder nur von PatientInnen allein oder zusammen mit Leistungserbringer genutzt.
- Die DiGA dient nicht der Primärprävention.

Inzwischen wurden zahlreiche DiGA in ein dafür eingerichtetes Verzeichnis aufgenommen – es zeigt sich jedoch, dass sich die Bereitschaft zur Rezeptierung von DiGA noch sehr zwischen den verschiedenen Fachrichtungen unterscheidet. Eine besonders hohe Bereitschaft zeigen Fachärztinnen und -ärzte der HNO-Heilkunde und der Neurologie, am geringsten ist die Bereitschaft in den Fächern Dermatologie und Augenheilkunde [3, 13].

Ziel dieser Arbeit ist die Identifikation von DiGA im DiGA-Verzeichnis, die der Behandlung von Erkrankungen aus dem HNO-Bereich dienen und Beschreibung der den jeweiligen DiGA zugrunde liegenden Evidenz.

## Material und Methoden

Entsprechend des Ziels dieser Arbeit wurden zunächst das DiGA-Verzeichnis analysiert und Anwendungen identifiziert, deren Indikationsbereich eine Erkrankung aus dem HNO-Bereich betrifft. Dabei wurden sowohl Anwendungen mit in die weitere Auswertung eingeschlossen, die für Erkrankungen ausschließlich des HNO-Bereichs gedacht sind (wie z. B. Anwendungen zur Behandlung von Tinnitus), aber auch Anwendungen, die für mehrere Erkrankungen erstellt wurden und unter anderem Erkrankungen aus dem HNO-Bereich betreffen (wie z. B. Anwendung zur Verhaltenstherapie nach bösartigen Erkrankungen). Zusätzlich wurden Anwendungen in die Auswertung einbezogen, die Erkrankungen behandeln, die neben anderen Fachrichtungen auch von Ärzten aus dem Bereich der HNO-Heilkunde behandelt werden (wie z. B. der Insomnie). Für die Auswahl der Anwendungen wurden auch die jeweiligen ICD-Codes, bei denen die DiGA angewendet werden soll, berücksichtigt.

**Figure 1:**
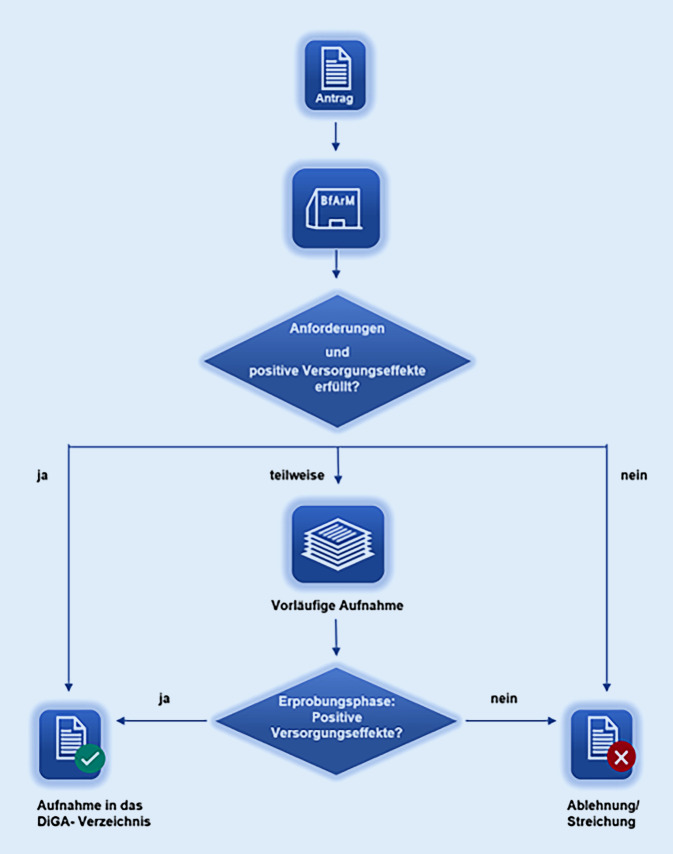

Neben Anwendungen, die entweder vorläufig oder dauerhaft in das Verzeichnis aufgenommen wurden, sind auch Anwendungen eingeschlossen worden, die nach einer vorläufigen oder dauerhaften Aufnahme aktuell gestrichen sind (Abb. 1). Bei jeder der identifizierten Anwendungen erfolgt zunächst eine Beschreibung des jeweiligen Anwendungsbereichs und anschließend eine Analyse der zugrunde liegenden Evidenz. Das Level der zugrunde liegenden Evidenz wurde nach den Empfehlungen des Oxford Centre for Evidence-Based Medicine für therapeutische Studien angegeben (Tab. 1; [4]).

**Table 1:** 

Level	Therapiestudien
1a	Systematische Übersichtsarbeit von randomisierten kontrollierten Studien (RCT)
1b	Einzelne RCT
1c	„All or none“, alle Patienten verstarben vor Therapie
2a	Systematische Übersichtsarbeit von Kohortenstudien
2b	Einzelne Kohortenstudie
2c	„Outcomes“-Forschung
3a	Systematische Übersichtsarbeit von Fall-Kontroll-Studien
3b	Einzelne Fall-Kontroll-Studie
4	Fallserien
5	Expertenmeinung

## Resultate

**Figure 2:**
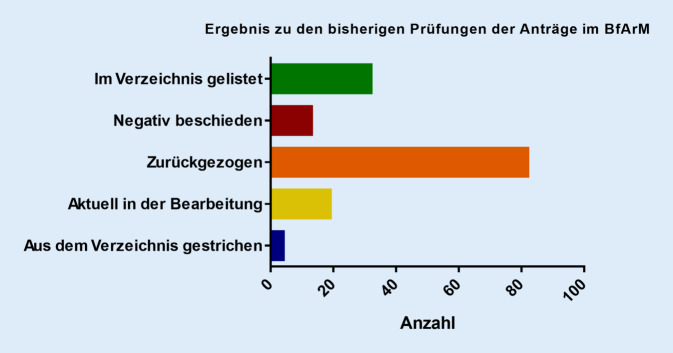

In Abb. 2 sind die Ergebnisse der Prüfungen von allen bisher eingereichten DiGA, die auch die Grundlage für unsere Auswertung bilden, dargestellt. Unter Anwendung der beschriebenen Einschlusskriterien wurden insgesamt sechs DiGA identifiziert, die im Weiteren entsprechend untersucht werden. Zwei DiGA sind für die Verhaltenstherapie bei einem Tinnitus vorgesehen, eine DiGA zur Rauchentwöhnung, eine DIGA zur Behandlung von schädlichem Alkoholkonsum, eine DiGA zur Verhaltenstherapie bei Insomnien und eine DiGA zur Linderung psychischer und psychosomatischer Folgen von Diagnose und Therapie verschiedener Malignome (Tab. 2).

**Table 2:** 

	Kalmeda	Meine Tinnitus-App	NichtraucherHelden-App	Somnio	Mika	Vorvida
Benötigte Hardware	–	–	–	–	–	–
Anwendungsdauer	90 T	10 W	90 T	90 T	4 W	90 T
Mindestanwendungsdauer	12 M	10 W	–	90 T	4 W	90 T
Höchstanwendungsdauer	12 M	–	–	–	–	–
HNO-spezifische Indikation	H93.1	H93.1	F17.2	F51.0	C00-14, C30-33, C43, C44	F10.1, F10.2
Verschreibungspreis	203,97 €	449,00 €	329,00 €	224,99 €	499,00 €	192,01 €
Zusatzkosten	–	–	–	–	–	–
Altersgruppe	Ab 18 J	Ab 18 J	Ab 18 J	Ab 18 J	Ab 18 J	Ab 18 J.
Vorl. Aufnahme	–	×	×	–	–	–
Dauerhafte Aufnahme	×	–	–	×	–	×
Evidenzniveau	1b	4	4	1b	1b	1b

*T* Tage, *W* Wochen, *M* Monate, *J* Jahre

### Kalmeda Tinnitus-App

Die Behandlung eines Tinnitus orientiert sich an der aktuellen S3-Leitlinie – wesentlicher Pfeiler der Behandlung ist eine kognitive Verhaltenstherapie (KVT), die mit einer hohen zugrunde liegenden Evidenz empfohlen werden [10, 17]. Die Empfehlung schließt auch internetbasierte Methoden ein, also auch DiGA. Die Kalmeda Tinnitus-App (mynoise GmbH, Duisburg, Deutschland) wurde in einer offenen, kontrollierten und randomisierten Studie, die den Effekt der Anwendung zwischen einer Interventionsgruppe und einer Kontrollgruppe, die erst nach einer dreimonatigen Wartezeit die Anwendungen nutzen konnte, untersucht. Primärer Outcome-Parameter war der Effekt auf die Belastung durch den Tinnitus (Tinnitus-Fragebogen von Göbel und Hiller), sekundäre Outcome-Parameter die Veränderung von Tinnitus-Belastung, Depressionsneigung, Stresserleben und Selbstwirksamkeit. Insgesamt wurden 187 PatientInnen in die Studie eingeschlossen, und alle Parameter in der Interventionsgruppe zeigten im Gegensatz zur Kontrollgruppe statistisch signifikante Verbesserungen.

### Meine Tinnitus-App – das digitale Tinnitus-Counseling

Anders als die Kalmeda Tinnitus-App stellt die DiGA Meine Tinnitus-App (Sonorem GmbH, Hamburg, Deutschland) ein digitales Angebot für das Counseling von PatientInnen mit Tinnitus im Rahmen der Erstversorgung nach Untersuchung durch den zuständigen Arzt dar. Durch die Vermittlung tinnitusspezifischer Multimediainhalte soll eine Aufklärung stattfinden und eine Basis für weitere therapeutische Interventionen geschaffen werden. Aktuell ist diese DiGA nur vorläufig in das DiGA-Verzeichnis aufgenommen, und die Studien zum Nachweis eines positiven Effekts dieser Behandlungsmethode laufen noch. Grundlage für die vorläufige Aufnahme war eine Probedatensammlung von insgesamt 67 TeilnehmerInnen, die eine Verbesserung der Tinnitus-Belastung (ermittelt mittels Mini-TF-12) und der krankheitsbedingten Schwierigkeiten im Alltag (BVB-2000) belegte. Eine randomisierte kontrollierte Studie zur Bestätigung dieser Beobachtung rekrutiert aktuell PatientInnen.

### NichtraucherHelden-App

Tabakkonsum ist, gerade in der Kombination mit Alkoholabusus, der bedeutendste Risikofaktor für die Entstehung von Malignomen im Bereich der Schleimhäute von Mundhöhle, Pharynx und Larynx. Die S3-Leitlinie „Rauchen und Tabakabhängigkeit: Screening, Diagnostik und Behandlung“ empfiehlt verhaltenstherapeutische Gruppen- und Einzelinterventionen mit dem höchsten Empfehlungsgrad. Über die DiGA NichtraucherHelden-App (NichtraucherHelden GmbH, Stuttgart, Deutschland) sollen PatientInnen durch eine KVT beim anhaltenden Überwinden einer Nikotinabhängigkeit unterstützt werden [1, 22]. Auch diese DiGA ist aktuell vorläufig in das DiGA-Verzeichnis aufgenommen. Grundlage hierfür ist eine Pilotstudie, bei der an einem Kollektiv von 50 eingeschlossenen ProbandInnen nach einem Anwendungszeitraum von vier Monaten die 7‑Tage-Prävalenz der Rauchabstinenz gemessen wurde. Eine randomisierte kontrollierte Studie rekrutiert aktuell PatientInnen.

### Somnio

Entsprechend Leitlinienempfehlungen soll bei Erwachsenen jeden Lebensalters mit Insomnie eine KVT die erste Behandlungsoption sein [18, 19, 21]. Bei der DiGA Somnio (mementor DE GmbH, Leipzig, Deutschland) werden evidenzbasierte Inhalte zur KVT für Insomnie (KVT-I) vermittelt. Der positive Versorgungseffekt wurde im Rahmen einer randomisierten kontrollierten Studie evaluiert [15]. Im Interventionsarm wurde die DiGA genutzt und mit einer Wartelisten-Kontrollgruppe verglichen. Es zeigte sich eine signifikante Besserung von Insomnie Schweregrad Index, Beck Depression Inventory, Brief Symptom Inventory und SF-12 Health Survey in der Interventionsgruppe.

### Mika

Die DiGA Mika (Fosanis GmbH, Berlin, Deutschland) hat das Ziel, die psychischen und psychosomatischen Folgen von Diagnose und Therapie verschiedener Malignome zu lindern. PatientInnen sollen auf verschiedenen Themengebieten weitergebildet und zum Selbstmanagement befähigt werden und bekommen Werkzeuge an die Hand, um selbst Einfluss auf den Erkrankungsverlauf zu nehmen. In einer randomisierten kontrollieren Pilotstudie wurde eine Interventionsgruppe (Nutzung von Mika) mit einer Kontrollgruppe (Standardversorgung) verglichen – primärer Endpunkt war die psychische Belastung, gemessen mit dem PHQ‑9. Nach einem Zeitraum von 12 Wochen zeigte sich hier eine signifikante Verbesserung in der Interventionsgruppe. Aktuell ist die DiGA nicht verfügbar, da sie auf Antrag des Herstellers aus dem Verzeichnis gestrichen wurde. Derzeit rekrutiert eine weitere randomisierte kontrollierte Studie Patienten zum Nachweis eines positiven Versorgungseffekts an einem größeren Patientenkollektiv.

### Vorvida

Wie bereits ausgeführt stellt Alkoholkonsum einen Risikofaktor in der Entstehung von Malignomen im Bereich der Schleimhäute der Mundhöhle und des Pharynx dar, ist aber darüber hinaus ein globales Gesundheitsproblem mit unterschiedlichen schwerwiegenden Folgen. Es konnte im Vorfeld gezeigt werden, dass eine internetbasierte Selbsthilfetherapie zu einer Alkoholkonsumreduktion bei Erwachsenen führen kann [20]. Diese Interventionsart ist auch in der S3-Leitlinie „Screening, Diagnose und Behandlung alkoholbezogener Störungen“ entsprechend empfohlen. Vorvida (GAIA AG, Hamburg, Deutschland) ist ein internetbasierter Ansatz zur kognitiven Verhaltenstherapie mit dem Ziel, den Alkoholkonsum zu reduzieren. In einer randomisierten kontrollierten Studie wurde der Effekt an einer Population von 608 Erwachsenen untersucht und mithilfe verschiedener Endpunkte (selbstberichteter Alkoholkonsum, Trinkverhalten, Zufriedenheit) nach sechsmonatiger Studiendauer belegt [23].

## Diskussion

Im Rahmen der erfolgten Auswertung wurden insgesamt sechs DiGA mit direktem oder indirektem Bezug zur HNO-Heilkunde identifiziert, von denen drei dauerhaft und zwei vorläufig in das Verzeichnis aufgenommen wurden. Eine DiGA ist aktuell (Stand 05.10.2022) vom Hersteller zurückgezogen worden. Es wird insgesamt ersichtlich, dass sich vor allem Erkrankungen für eine Behandlung mit einer DiGA eignen, für die verhaltenstherapeutische Behandlungsansätze verfügbar sind bzw. eine Therapiemöglichkeit darstellen. Dies betrifft nicht nur DiGA, die im HNO-Bereich Anwendung finden, sondern auch DiGA anderer Fachdisziplinen (z. B. zur Therapie von Depressionen oder Angststörungen). Für alle DiGA, gerade für die dauerhaft aufgenommenen, liegen Studien eines hohen Evidenzlevels zugrunde – für die vorläufig aufgenommenen laufen aktuell Studien, mit denen ein ähnlich hohes Evidenzlevel erreicht werden kann. Einschränkend ist hier jedoch anzumerken, dass die Studienergebnisse, trotz Nachweis eines positiven Versorgungseffekts, nicht in jedem Fall in Zeitschriften mit Peer-Review-Verfahren veröffentlicht wurden.

Trotz des innovativen Verfahrens und der zum Zeitpunkt der Einführung weltweiten Einmaligkeit von „Apps auf Rezept“ gibt es jedoch auch Kritikpunkte an DiGA [5]. Kontrovers werden etwa die Kosten, die aus der Verschreibung von DiGA für die Krankenversicherungen entstehen, diskutiert. Eine Umfrage, die von Handelsblatt Inside unter den zwanzig größten gesetzlichen Krankenkassen durchgeführt wurde (darunter die AOK, TK, Barmer, DAK-Gesundheit und weitere kleinere Betriebskrankenkassen), kam zum Ergebnis, dass bei diesen Versicherungen, die insgesamt 62 Mio. Versicherte abdecken, innerhalb des ersten Jahres nach Einführung der DiGA 38.000 Verschreibungen bewilligt wurden. Hochgerechnet auf die Zahl aller gesetzlich Versicherter (73 Mio.) würde dies insgesamt 45.000 verschriebene und bewilligte DiGA in Deutschland ergeben. Zum damaligen Zeitpunkt betrug der Durchschnittspreis der aufgenommenen DiGA 402 €, sodass im ersten Jahr nach Einführung der DiGA die Kosten für die Krankenkassen bei ungefähr 18 Mio. € gelegen sind. Damit lagen die Verschreibungen und damit verbundenen Kosten noch unter einer Schätzung der Boston Consulting Group, die (auch unter der Annahme, dass DiGA häufig auch in mehreren aufeinanderfolgenden Quartalen verschrieben werden) von Kosten zwischen 100 und 200 Mio. € für die Jahre 2021 und 2022 ausging. Bis zum Jahr 2025 wurden sogar Kosten von über einer Milliarde € pro Jahr prognostiziert (bei konstanten Preisen der DiGA und einer Durchdringung zwischen ein und zwei Prozent) [2]. Aktuell ist es so, dass sich die Preise für DiGA in einer Spanne von minimal 119 € pro Quartal bis maximal 744 € pro Quartal bewegen. Die vorgestellten DiGA für Erkrankungen mit Bezug zum HNO-Bereich bewegen sich innerhalb einer Preisspanne von 203,97 € und 499,00 € und weisen damit ebenfalls eine erhebliche Preisspanne auf.

Es ist bemerkenswert, dass die Hersteller von DiGA im Erprobungsjahr der jeweiligen Anwendung die Wirksamkeit noch nicht nachweisen müssen, dennoch aber den Preis für die DiGA selbst bestimmen können. Dies wird wiederholt von den Spitzenverbänden der Krankenkassen kritisiert, von anderen Verbänden, wie etwa dem Spitzenverband Digitale Gesundheitsversorgung, aber verteidigt: Nur so sei es überhaupt möglich, dass kleinere Hersteller entsprechende Anwendungen entwickeln können, und somit werde Innovation gefördert. Dieses Erprobungsjahr wird tatsächlich von vielen Herstellern genutzt, Stand März 2022 waren zwei Drittel der gelisteten DiGA lediglich vorläufig in das DiGA-Verzeichnis aufgenommen. Im Umkehrschluss führt dies natürlich dazu, dass während dieses ersten Jahres Anwendungen verschrieben werden, denen noch nicht die notwendige wissenschaftliche Evidenz zugrunde liegt, sondern lediglich vorläufige Ergebnisse [12]. Möglich ist dies, weil DiGA als Medizinprodukte mit einer geringen Risikoklasse eingestuft werden (Risikoklassen I oder IIa, Tabelle 8), ein entsprechendes Vorgehen könnte man sich z. B. im Rahmen der Einführung von Medikamenten kaum vorstellen. Hier muss sicherlich auch beachtet werden, ob durch eine DiGA, deren Effektivität noch nicht ausreichend belegt ist, möglicherweise eine etablierte, auf entsprechende Evidenz basierende Therapieform verdrängt wird oder ob eine DiGA eine Ergänzung einer derartigen Therapieform darstellt. Während Zweiteres sicher eine günstige Situation darstellt, kann die erste Konstellation, auch wenn von der DiGA selbst nur ein geringes Risiko ausgeht, dennoch ein Schaden für den Patienten oder die Patientin entstehen. Die Ansprüche, die von den Gesetzgebern für den Nachweis eines positiven Versorgungseffekt gestellt werden, sind dennoch hoch, sodass Hersteller entweder nach vorläufiger Aufnahme ihrer Anwendung in das Verzeichnis diese nach Durchführung entsprechender Studien wieder zurückziehen mussten oder bereits während der Antragsphase aufgrund eines nicht hinreichenden Studiendesigns ihre Anträge überarbeiten oder zurückziehen mussten [11].

Zusammenfassend lässt sich sagen, dass das Digitale-Versorgung-Gesetz einige Neuerungen beinhaltet und gerade die Einführung der DiGA, also der „Apps auf Rezept“, zu diesem Zeitpunkt eine weltweite Einmaligkeit war und inzwischen sogar vielen Ländern als Vorbild dient. Gerade Start-ups nutzten diese neue Möglichkeit, und es wurden schnell DiGA für ganz unterschiedliche Indikationen und Erkrankungsbilder entwickelt, wobei sich schnell zeigte, dass sich gerade Erkrankungen mit verhaltenstherapeutischen Behandlungsmöglichkeiten gut für eine derartige Behandlungsform eigenen. Das mag auch dazu geführt haben, dass sich zwischen verschiedenen Fachrichtungen deutliche Unterschiede hinsichtlich der Verordnungshäufigkeit von DiGA zeigten.

Es gibt Erkrankungen aus dem HNO-Bereich, die sich gut für DiGA-vermittelte und sogar leitlinienkonforme Therapieformen eignen. In der vorliegenden Arbeit werden insgesamt fünf unterschiedliche DiGA für Erkrankungen aus dem HNO-Bereich identifiziert, von denen aktuell (Stand 05.10.2022) vier tatsächlich verfügbar sind und teilweise zu den mit am häufigsten verordneten DiGA insgesamt gehören [7]. Die Evidenz hinter diesen DiGA basiert entweder auf randomisierten kontrollierten Studien, die bereits beendet wurden, oder auf Studien, oder aktuell noch rekrutieren.

Trotz des innovativen Aspekts der DiGA und des nachgewiesenen Versorgungseffekts einzelner DiGA gibt es jedoch verschiedene Kritikpunkte, etwa, was die Preise für die Verordnung von DiGA angeht. Möglicherweise sind dies Erklärungen, warum DiGA von vielen Ärzten noch nicht akzeptiert bzw. zumindest nicht häufig verordnet werden [6].

Die noch junge Form der Therapie mittels digitaler Gesundheitsanwendungen wird zukünftig sicherlich noch durch neue Regelungen oder Anpassungen verändert, um so auf bisherige Kritikpunkte zu reagieren. Dennoch stellt dieser Bestandteil des Digitale-Versorgung-Gesetzes eine tatsächliche Innovation dar, die vielen Patienten eine entsprechende Therapie ermöglicht kann. Gerade in Zeiten des Mangels an Ärzten (und Psychotherapeuten), noch verstärkt etwa in ländlichen Regionen, können DiGA eine zunehmend wichtigere Rolle spielen.
